# Artificial intelligence‐enabled cytopathological interpretation of fluorescence microscopy images via a multimodal large language model

**DOI:** 10.1002/qub2.70042

**Published:** 2026-04-30

**Authors:** Atakan Tevlek, Ozgecan Ocakcı, Beyza Emeksiz

**Affiliations:** ^1^ Department of Medical Biology Faculty of Medicine Atilim University Ankara Türkiye; ^2^ Undergraduate Program Faculty of Medicine Atilim University Ankara Türkiye

**Keywords:** apoptosis, artificial intelligence, cytopathology, fluorescence microscopy, image analysis, multimodal large language model

## Abstract

Quantitative analysis of fluorescence microscopy images remains a significant challenge in cytopathological assessments, as subjective interpretation and inter‐observer variability hinder reproducibility and standardization of classifications. In this study, an artificial intelligence‐assisted analytical framework utilizing a multimodal large language model (MLLM) was employed to classify cytotoxic responses in acridine orange/propidium iodide‐stained MCF‐7 cells subjected to doxorubicin treatment for 24 h. A total of 500 fluorescence images, collected from five experimental groups including a control and four dose‐dependent treatment conditions, were examined to identify distinct morphological indicators of viability, necrosis, and apoptosis. Ground truth annotations were established using a standardized 15‐parameter cytopathological evaluation checklist, conducted by domain experts. The MLLM was subsequently tasked with interpreting the same dataset and generating structured cytopathological outputs aligned with expert‐defined criteria. Receiver operating characteristic analysis demonstrated moderate discriminative performance for viable cells (area under the curve [AUC] = 0.68) and strong discriminative performance for necrotic cells (AUC = 0.84), reflecting reliable identification of overt necrotic morphology. However, early and late apoptotic states showed poor separability (AUC = 0.52–0.55) and were inconsistently distinguished, which can be attributed to inadequate spatial–contextual inference within the model. The entire dataset was processed within 2 h, demonstrating a significant enhancement in analytical throughput relative to manual evaluation. These findings indicate that MLLM‐driven cytopathological analysis may serve as an intermediate automation layer between traditional microscopic assessment and fully autonomous deep learning‐based image processing systems, facilitating scalable, standardized, and semi‐autonomous interpretation of fluorescence imaging data.

## 1 INTRODUCTION

Microscopy‐based cellular imaging plays a crucial role in understanding the structural and functional responses of cells to physiological and pathological stimuli [1]. Under physiological conditions, cells exhibit morphology that aligns with their lineage and functional state; however, in response to stress, injury, or oncogenic transformation, they undergo distinct alterations in nuclear and cytoplasmic architecture that are detectable through microscopy and serve as key diagnostic indicators [2]. Identifying these morphological alterations is particularly important for elucidating the mechanisms of cell death, a characteristic feature of numerous pathological processes, including cancer, neurodegenerative disorders, ischemic injuries, autoimmune diseases, and infectious pathologies [3]. Among the various types of cell death, apoptosis and necrosis are two fundamental and well‐characterized mechanisms, and their distinction holds critical value for both basic research and diagnostic applications [4]. To this end, fluorescence microscopy coupled with dual staining using acridine orange (AO) and propidium iodide (PI) has been widely adopted as a reliable method for distinguishing viable, apoptotic, and necrotic cells, owing to its sensitivity to membrane permeability and nuclear morphology [5]. AO binds to nucleic acids in both viable and dying cells, emitting green fluorescence, whereas PI selectively penetrates cells with compromised membranes, producing red fluorescence [6]. This dual‐staining approach enables visual identification of distinct stages of cell death; however, its interpretation traditionally relies on manual inspection, which is time‐consuming, subjective, and dependent on expert judgment [7].

Over the past 2 decades, artificial intelligence (AI) has been increasingly integrated into biomedical image analysis to address the inherent challenges of manual cytopathological evaluation, including the time‐consuming nature of image screening, observer variability, limited reproducibility, and the dependency on expert‐level training [8]. Traditional machine learning and, more recently, convolutional neural networks (CNNs) have demonstrated efficacy in tasks including cell segmentation, counting, and phenotypic categorization in brightfield or phase‐contrast microscopy [9, 10, 11, 12, 13]. Nevertheless, these systems generally necessitate extensive annotated datasets, are frequently task‐specific, and provide restricted flexibility or interpretability [14]. Additionally, fluorescence microscopy‐based cell recognition and classification have remained relatively underexplored in comparison with brightfield applications [15].

The emergence of multimodal large language models (MLLMs), including Claude Sonnet, ChatGPT (OpenAI), DeepSeek, and Gemini (Google DeepMind) has introduced a revolutionary paradigm in AI by enabling the simultaneous interpretation of textual and visual data within a cohesive framework [16, 17]. Unlike traditional deep learning architectures such as CNNs, MLLMs are capable of flexible instruction‐based reasoning and can generate structured outputs aligned with domain‐specific criteria [18]. These models successfully integrate image recognition with natural language generation, enabling them to model expert decision‐making via interactive prompts in a reproducible and identifiable form [19]. Initially developed for general tasks such as visual question answering and multimodal document understanding, MLLMs are now being rapidly adapted for biomedical domains owing to their versatility and accessibility [20]. For instance, GPT‐4V has been applied to chest radiographs for diagnostic purposes, while DeepSeek‐R1 has shown promise in multimodal clinical reasoning tasks, including surgical workflow interpretation and medical education simulations [21, 22]. Comparative analyses have also evaluated models such as GPT‐4o, Claude 3.5 Sonnet, and Gemini 1.5 Pro in dermatological diagnosis, demonstrating diagnostic performance comparable to, and in some complex cases exceeding, that of board‐certified dermatologists, thereby highlighting their potential as supplementary tools in clinical dermatology [23]. Similarly, Claude 3.5 Sonnet has been assessed for assigning TNM classifications in pancreatic cancer based on CT findings, showing a significant improvement in T‐factor accuracy over GPT‐4 Turbo, yet still requiring expert oversight due to persistent misclassifications and model‐specific limitations [24]. In ophthalmology, GPT‐4o has been utilized for glaucoma detection from color fundus photographs using the retinal fundus glaucoma challenge (REFUGE) dataset, achieving 90% diagnostic accuracy without prior fine‐tuning, suggesting the potential of large language models to support ophthalmic diagnosis even in data‐scarce settings [25]. Furthermore, GPT‐4o has been applied to classify blood cell morphologies from microscopic images provided by the American Society of Hematology, demonstrating 88% accuracy for normal cells and 54% for abnormal cells, suggesting its potential as an auxiliary tool in hematological diagnostics [26].

Despite their expanding use in fields such as radiology, dermatology, oncology, and ophthalmology, MLLMs have seen limited application in fluorescence‐based cytopathology—particularly in analyzing AO/PI‐stained cellular images. However, recent progress indicates their growing capability in complex biomedical imaging tasks, including microscopy‐based cellular morphology. Among currently available MLLMs, Claude 3.7 Sonnet was selected for the primary experiments based on its demonstrated strengths in structured instruction‐following, stable multimodal reasoning, and comparatively reproducible schema‐aligned outputs under low‐temperature settings. In contrast to models primarily optimized for conversational or open‐ended visual question answering (e.g., GPT‐4o) or long‐context document understanding (e.g., Gemini 1.5 Pro), Claude Sonnet has shown improved robustness in prompt‐controlled analytical tasks requiring strict adherence to predefined evaluation criteria and output formats. These characteristics were particularly critical for the present study, which relies on a fixed expert‐defined 15‐parameter cytopathological evaluation checklist and requires reproducible, parameter‐wise interpretation rather than free‐form descriptive responses. Building on this trend, the present study evaluates the performance of Claude Sonnet, a state‐of‐the‐art MLLM, in interpreting AO/PI‐stained fluorescence microscopy images of doxorubicin‐treated MCF‐7 cells. Using a standardized 15‐parameter cytopathological evaluation checklist, model outputs were compared with expert evaluations across parameters such as total cell count, viability, apoptotic and necrotic indices, and nuclear/cytoplasmic morphology. Quantitative validation included precision, recall, F1‐score, and area under the curve (AUC), focusing on apoptotic and necrotic detection. Intra‐model repeatability was also assessed. This comprehensive evaluation aimed to demonstrate the feasibility, reproducibility, and diagnostic potential of MLLMs in fluorescence‐based cytopathological screening as scalable tools for AI‐assisted biomedical imaging workflows.

## 2 RESULTS

The cytopathological evaluation of AO/PI‐stained MCF‐7 cells treated with increasing concentrations of doxorubicin (0–4.0 μmol/L) demonstrated a clear dose‐dependent pattern in cellular responses. Across all treatment groups, fluorescence microscopy images revealed progressive alterations in cell morphology and staining characteristics, reflecting a gradual shift from predominantly viable cells to apoptotic and necrotic phenotypes. Green fluorescence indicated intact viable cells, whereas orange to red fluorescence marked cells undergoing apoptosis or necrosis. These changes became increasingly evident at higher drug concentrations, as shown in representative images in Figure 1. The emergence and intensification of distinct morphological hallmarks—including chromatin condensation, nuclear fragmentation (NF), membrane blebbing (MB), and nuclear swelling (NS)—were observed with increasing doxorubicin concentrations, consistent with apoptotic and necrotic morphological patterns.

**FIGURE 1:**
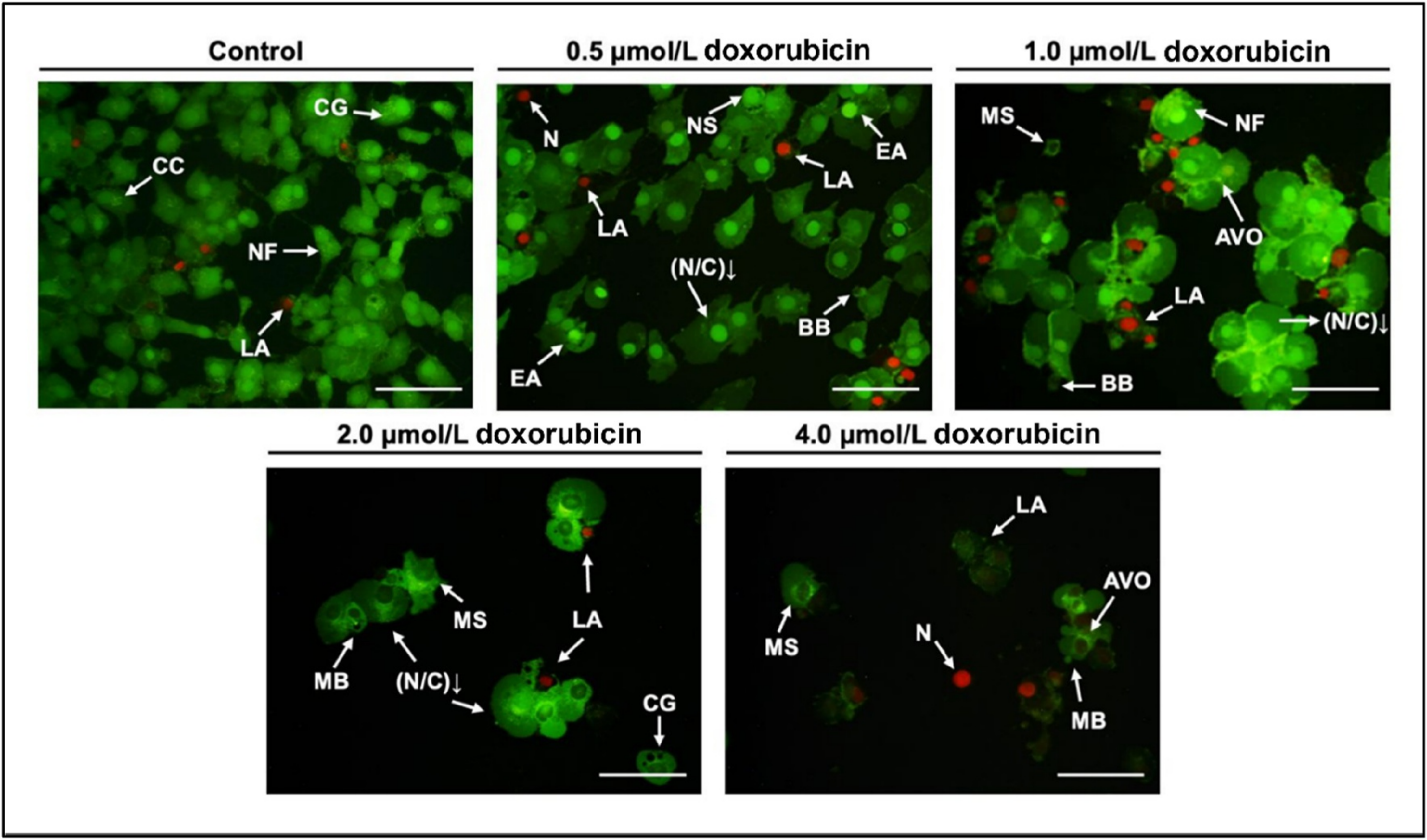
Representative fluorescence microscopy images of acridine orange/PI‐stained MCF‐7 cells exposed to increasing concentrations of doxorubicin (0–4.0 μmol/L), illustrating dose‐dependent cytopathological changes as identified by human experts. Green fluorescence indicates viable cells, while red fluorescence marks apoptotic or necrotic cells. Annotated morphological features include chromatin condensation (CC+), cytoplasmic granularity (CG), acidic vesicular organelles (AVO), membrane blebbing (MB), nuclear fragmentation (NF), nuclear swelling (NS), blebbing (BB), morphological shift (MS), early apoptosis (EA), late apoptosis (LA), necrosis (N), and changes in the nucleus‐to‐cytoplasm ratio (N/C). With increasing drug concentration, a higher prevalence of nuclear and membrane abnormalities was observed from 1.0 μmol/L onward. Scale bar: 100 μm.

Notably, even at low concentrations such as 0.5 μmol/L and 1.0 μmol/L, distinct cytopathological features could be readily detected. Morphological indicators of early apoptosis—including chromatin condensation, membrane blebbing, and cytoplasmic granularity (CG)—were prominently observed, despite a considerable number of viable cells still being present. At these doses, apoptotic features were observed in the absence of widespread necrosis, enabling visualization of transitional morphological states. However, as the doxorubicin concentration increased to 2.0 μmol/L and 4.0 μmol/L, the proportion of viable cells dropped sharply. At these higher doses, fluorescence microscopy fields were dominated by fragmented, disintegrated, or fully necrotic cells, with markedly reduced total cell numbers. The scarcity of intact cell structures and the prevalence of diffused red‐stained debris limited the identification of discrete cytopathological markers. Accordingly, overall cellular integrity and countable cell availability were substantially reduced at higher concentrations.

In addition to these qualitative morphological observations, a structured comparison was carried out between expert evaluations and MLLM outputs, as described in the Materials and Methods section (Section 5). This comparative analysis was quantitatively summarized to highlight convergence and divergence in detecting key cytopathological parameters across treatment groups, providing a more comprehensive picture of interpretive consistency. The graphical representation of these results is presented in the following figure. For graphical clarity, Figure 2 displays data derived from a representative subset of 10 randomly selected images per group, selected from the full dataset of 100 images per group used in the overall analysis.

**FIGURE 2:**
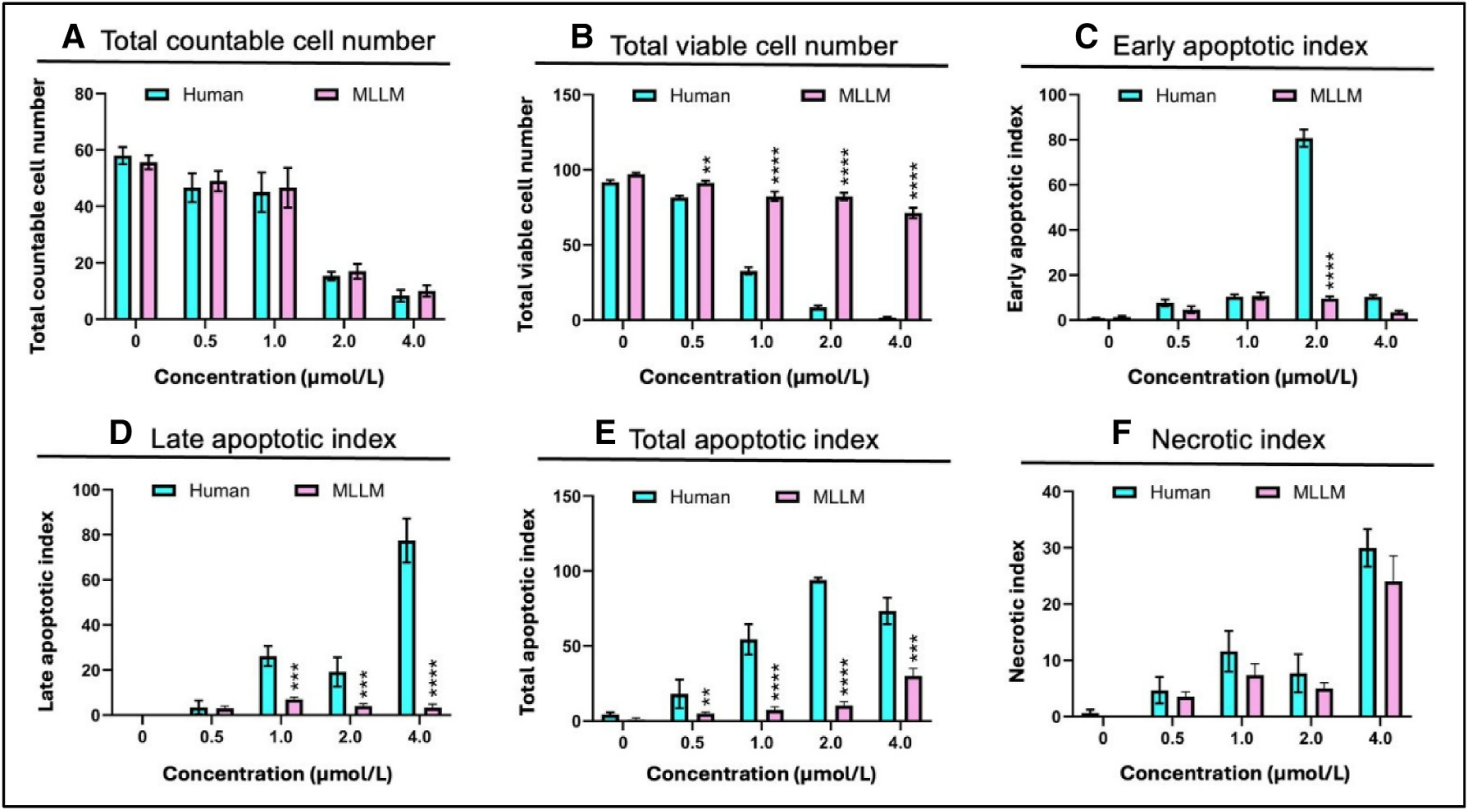
Comparative analysis of six key cytopathological parameters evaluated by human experts and a MLLM in acridine orange/PI‐stained MCF‐7 cells exposed to increasing concentrations of doxorubicin (0–4.0 μmol/L). (A) Total countable cell number. (B) Total viable cell number. (C) Early apoptotic index. (D) Late apoptotic index. (E) Total apoptotic index, and (F) Necrotic index. Expert (human) and AI (MLLM) assessments are shown as mean ± standard deviation (*n* = 10 per group). Although MLLM performed comparably to experts in total cell count and necrotic detection, notable discrepancies were observed in apoptotic indices, particularly at intermediate and high drug concentrations. Statistical significance is indicated by asterisks and denotes comparisons between each treated group and the untreated control group (0 μmol/L doxorubicin): *p < 0.05, **p < 0.01, ***p < 0.001, ****p < 0.0001 versus control. MLLM, multimodal large language model.

The total number of countable cells per image was consistent between human experts and MLLM analyses across all concentrations as shown in Figure 2A. Both approaches identified approximately 60 cells on average per field. This strong concordance suggests that MLLMs can effectively detect and count cells under fluorescence microscopy conditions, irrespective of doxorubicin concentration. Assessment of viable cell populations (VI %) further underscored the divergence between human experts and the MLLM as shown in Figure 2B. Human expert evaluations revealed a sharp decline in viability beginning at 1.0 μmol/L, with values dropping below 10% at the highest dose (4.0 μmol/L), reflecting the cytotoxic impact of doxorubicin. Although the MLLM successfully captured the overall downward trend, it consistently overestimated viability across all treatment groups. This discrepancy likely stems from the model’s limited ability to distinguish between truly viable cells and those exhibiting early‐stage cytoplasmic or nuclear alterations that precede apoptosis. Another possible reason could be the model’s tendency to rely heavily on the presence of intact green fluorescence, without fully interpreting subtle structural deformations that human evaluators recognize as pre‐apoptotic.

When it comes to early apoptotic evaluation shown in Figure 2C, both the MLLM and human experts demonstrated a high degree of consistency at lower doxorubicin concentrations (control–1.0 μmol/L), where apoptotic features such as chromatin condensation and membrane blebbing were relatively easier to distinguish. At these doses, early apoptotic events were clearly identifiable, allowing the model to closely replicate expert assessments. However, at 2.0 μmol/L, expert analysis revealed a sharp rise in early apoptotic cells—reaching nearly 80%—which the MLLM failed to reflect, instead reporting its peak at 1.0 μmol/L. At higher concentrations (2.0–4.0 μmol/L), early apoptotic indices decreased in expert evaluations, coinciding with a progressive increase in late apoptotic and necrotic phenotypes as observed by AO/PI fluorescence microscopy. Under these conditions, extensive structural disruption and reduced cellular integrity limited the visibility of discrete early apoptotic morphology. In parallel, discrepancies between human experts and MLLM assessments became more pronounced at these concentrations. Regarding late‐stage apoptotic assessment (Figure 2D), human experts identified a progressive increase in late apoptotic cells, with the highest incidence observed at 4.0 μmol/L, where nearly 80% of cells were classified as late apoptotic. In contrast, the MLLM consistently reported low late apoptotic indices (< 10%) across all concentrations, indicating substantial divergence between expert and model‐based evaluations at advanced stages of cell death.

In efforts to quantify apoptotic burden regardless of stage, total apoptotic index (TAI %)—the combined proportion of early and late apoptotic cells—was also evaluated and shown in Figure 2E. Expert assessments indicated a pronounced peak at 2.0 μmol/L, where nearly all observable cells (approaching 100%) exhibited apoptotic morphology. In contrast, the MLLM demonstrated a more gradual and flattened response across the dose range, with consistently lower TAI values and no detection of apoptotic cells in the control group. In parallel, discrepancies between human expert and MLLM assessments observed in early and late apoptotic indices were also reflected in the total apoptotic index. At higher concentrations, the increasing prevalence of structurally compromised cells coincided with reduced visibility of discrete apoptotic features under AO/PI fluorescence microscopy. Unlike apoptotic parameters, the assessment of necrotic cell death showed a high level of agreement between human expert evaluations and MLLM outputs, as presented in Figure 2F. Both approaches demonstrated a clear dose‐dependent increase in necrotic index (NI %), with only minor quantitative differences across concentrations.

To further interrogate the model’s interpretative capacity—particularly in the presence of overlapping apoptotic and necrotic phases—side‐by‐side heatmaps were constructed to compare human expert‐based and MLLM‐based detailed qualitative cytopathological evaluations across doxorubicin concentrations ranging from 0 to 4.0 μmol/L, where both early and late apoptotic features coexist at varying intensities. This approach enabled a direct visual comparison of detection trends, consistency across doses, and divergences in sensitivity between the human and machine assessments. By applying a unified color scale, interpretive patterns could be rapidly evaluated at both the intra‐dose (within each group) and inter‐dose (across doses) levels, as shown in Figure 3.

**FIGURE 3:**
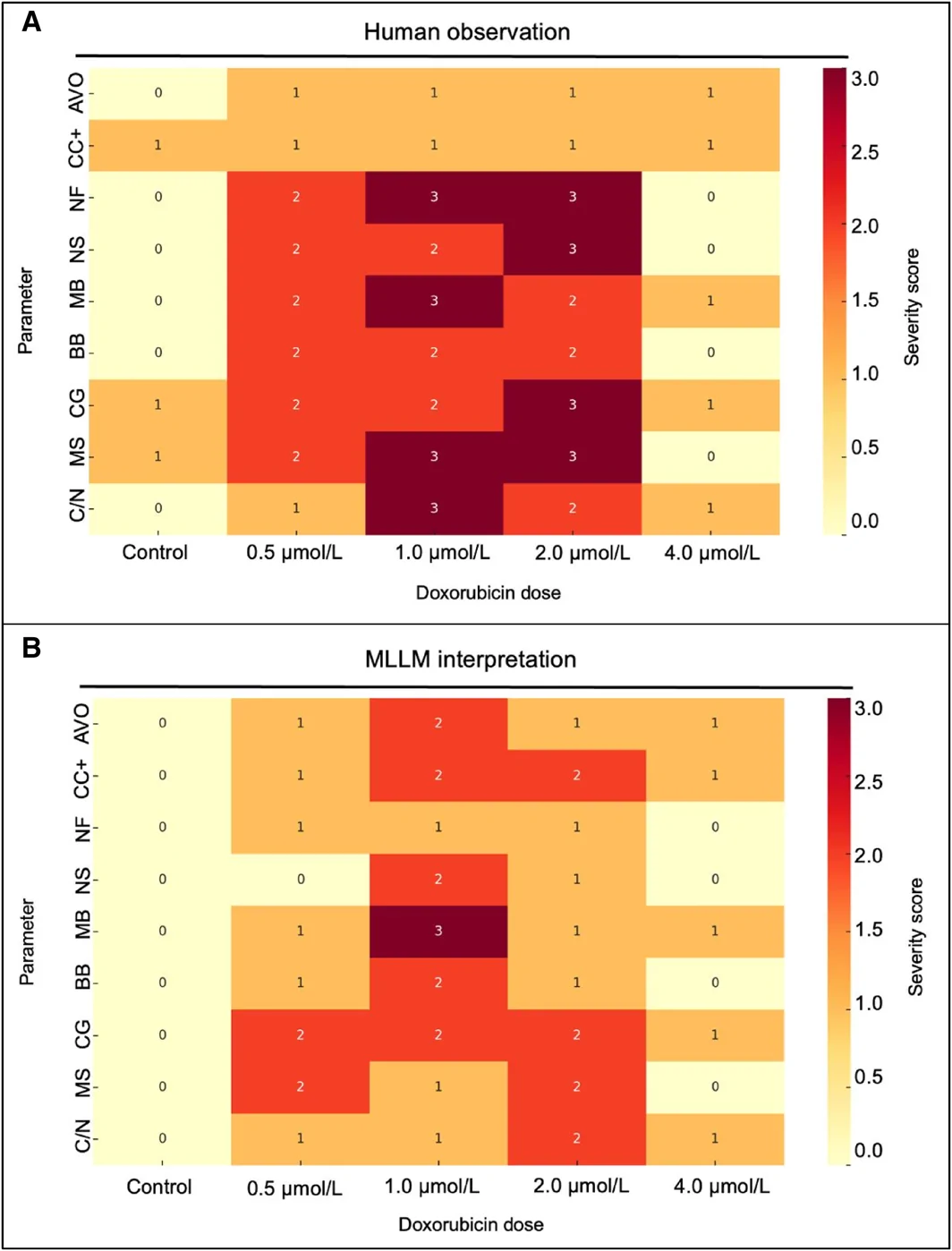
Comparative heatmap representation of cytopathological parameter severity across doxorubicin concentrations. (A) Heatmap constructed from human expert‐assessed qualitative evaluations converted into standardized severity scores (0: None, 1: Minimal, 2: Moderate, 3: Extensive). (B) Heatmap based on MLLM‐generated predictions for the same parameters using the identical scoring format. Both panels use a unified color scale (YlOrRd) to allow direct visual comparison across five treatment groups (control, 0.5 μmol/L, 1.0 μmol/L, 2.0 μmol/L, and 4.0 μmol/L doxorubicin), highlighting dose‐dependent morphological alterations and interpretive divergences between human and machine assessments. AVO, acidic vesicular organelles; BB, blebbing; C/N: cytoplasm‐to‐nucleus ratio; CC+, condensed chromatin; CG, cytoplasmic granularity; MB, membrane blebbing; MS, morphological shift; NF, nuclear fragmentation; NS, nuclear swelling.

The heatmap presented in Figure 3A demonstrates a clear dose‐dependent increase in cytopathological severity from 0.5 μmol/L to 2.0 μmol/L doxorubicin. Apoptotic and necrotic markers such as NF, NS, MB, and morphological shift (MS) progressively intensify, aligning with expected cellular responses to chemotherapeutic stress. Notably, at the highest concentration of 4.0 μmol/L, several apoptotic parameters—including NF, NS, blebbing (BB), and MS—drop to a severity score of 0. This observation reflects the biological reality that high‐dose doxorubicin primarily induces rapid necrotic death, resulting in extensive structural collapse and the disappearance of intact cellular morphology. In such conditions, apoptotic features become unobservable not due to their theoretical absence but because the cells have disintegrated beyond morphologically recognizable states under fluorescence microscopy. Meanwhile, parameters such as CG, acidic vesicular organelles (AVO), and condensed chromatin (CC+) persist at minimal levels (score 1), likely representing residual staining in fragmented or necrotic debris. These low‐level traces suggest that certain morphological indicators may remain detectable even under severe cytotoxic degradation, although their diagnostic specificity may be limited.

The heatmap presented in Figure 3B illustrates a partially dose‐responsive pattern in cytopathological scoring based on MLLM‐generated predictions. In the control and 0.5 μmol/L groups, most parameters—including CG, CC+, and MS—receive scores of 0 or 1, indicating limited model sensitivity to subtle morphological features that are typically detected by human experts. At intermediate concentrations (1.0–2.0 μmol/L), the model begins to assign moderate scores across several parameters, including MB, CG, and CC+. However, critical apoptotic markers—such as NF, NS, and BB—remain consistently under detected, despite their clear presence in expert evaluations. This underrepresentation suggests that the model struggles to identify nuanced or transitional apoptotic changes that require spatial and contextual interpretation. In contrast to expert findings, the model tends to assign moderate scores across multiple parameters even at 4.0 μmol/L, where apoptotic morphology is largely absent due to necrotic disintegration. This mismatch may stem from the model’s inability to distinguish between structured apoptotic signals and background noise or residual debris, highlighting a lack of context‐aware biological reasoning under extreme cytotoxic conditions.

To better contextualize the discrepancies observed between expert and MLLM‐based evaluations, a categorical analysis was conducted to quantify convergence and divergence patterns across all 45 parameter‐dose combinations (9 parameters × 5 doxorubicin doses). Figure 4 summarizes this comparison into five interpretative classes: full convergence, slight overestimation, slight underestimation, strong overestimation, and strong underestimation. This classification provides a broader understanding of how frequently, and in which direction, the model deviates from expert interpretation.

**FIGURE 4:**
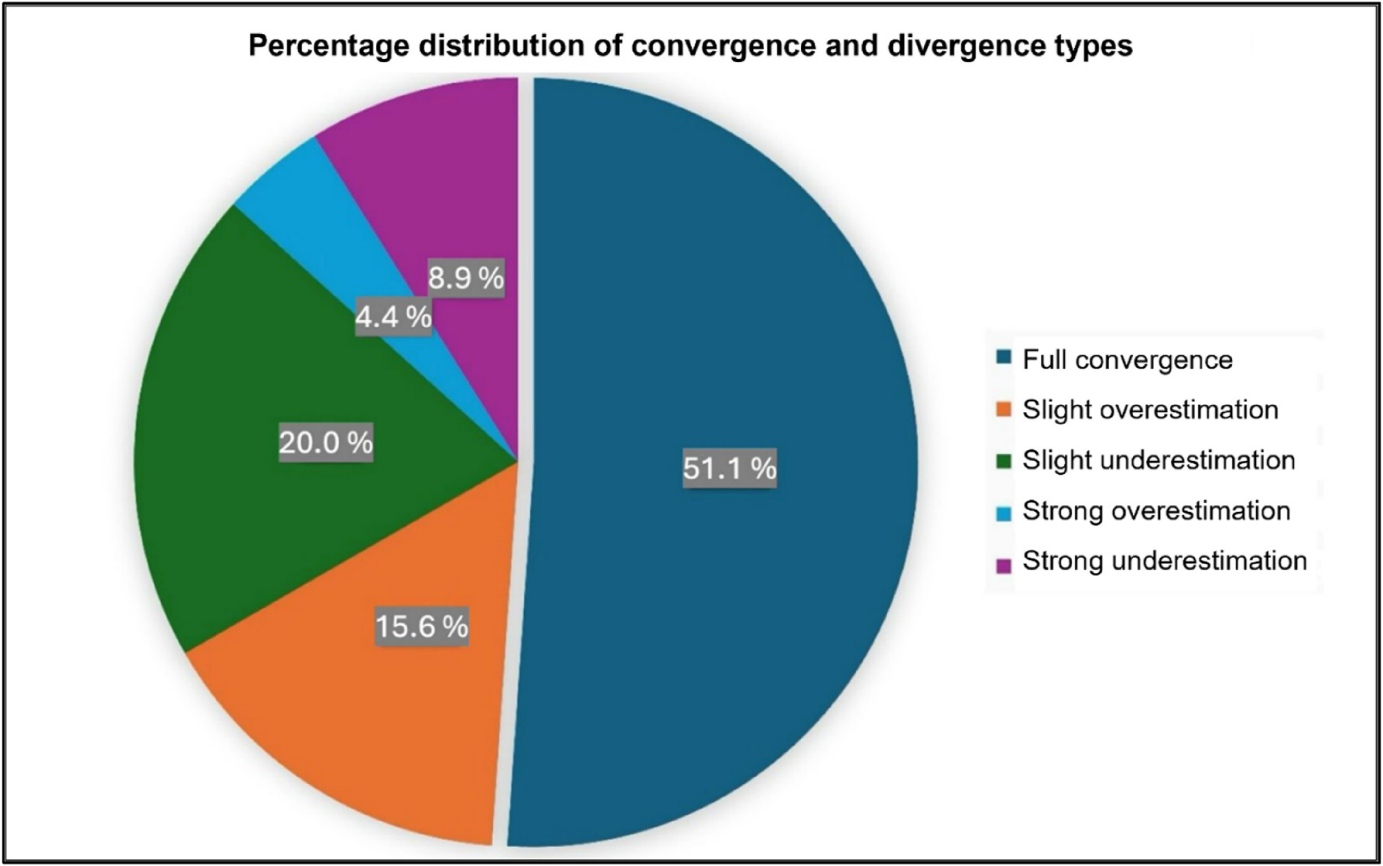
Percentage distribution of convergence and divergence between human and MLLM evaluations. The pie chart illustrates the outcome distribution across a total of 45 pairwise comparisons, based on 9 cytopathological parameters assessed at 5 different doxorubicin concentrations. Scoring results were categorized into five interpretative relationship types: full convergence (51.1%), slight overestimation (15.6%), slight underestimation (20.0%), strong overestimation (4.4%), and strong underestimation (8.9%). These proportions reflect the extent and direction of model–expert alignment, providing insight into the consistency and interpretive bias of the MLLM across varying morphological contexts. MLLM, multimodal large language model.

Among these, full convergence accounted for 51.1% of comparisons, indicating that in more than half of the cases, the MLLM successfully replicated expert‐assigned severity scores. This finding highlights the model’s general capacity to align with human expert‐level evaluations in fluorescence‐based cytopathological assessment. However, the remaining 48.9% of cases revealed varying degrees of divergence. Notably, underestimation emerged as the predominant error type. Slight underestimation (20.0%) was especially common at intermediate doxorubicin concentrations (1.0–2.0 μmol/L), where transitional apoptotic features such as chromatin condensation, NS, and blebbing are typically present. This trend suggests that the model often struggles to detect subtle morphological changes requiring nuanced spatial interpretation. Strong underestimation (8.9%) further reflects the model’s limitations in recognizing advanced but morphologically degraded apoptotic events. Conversely, overestimation occurred less frequently. Slight overestimation (15.6%) and strong overestimation (4.4%) together comprised a smaller portion of the distribution, indicating a relatively conservative interpretive pattern. Although this conservatism may reduce false‐positive detections, it potentially limits sensitivity—particularly in identifying early or ambiguous apoptotic cues. To further quantify agreement between expert annotations and MLLM outputs, Cohen’s kappa coefficient was calculated. The resulting *κ* value of 0.29 indicates a fair level of agreement, highlighting both the model’s potential to approximate human judgment and the need for refinement in interpreting morphologically ambiguous features [27]. A subset of 50 images (10 per treatment group) was re‐evaluated after a 72‐h interval to assess intra‐model repeatability. Identical final four‐class cell state classifications (viable, early apoptotic, late apoptotic, and necrotic) were obtained in 92.5% of cases, supporting the model’s internal consistency under controlled input conditions.

To better understand how these interpretive limitations translate into classification performance, we analyzed the model’s predictions using a confusion matrix and corresponding statistical metrics. Figure 5A presents the confusion matrix that combines the MLLM’s predicted classifications with expert annotations across four cytopathological states: viable, early apoptotic, late apoptotic, and necrotic. Accurate predictions are concentrated along the diagonal (highlighted with dashed lines), whereas off‐diagonal entries represent misclassifications. The model accurately identifies viable (21/25) and necrotic (17/25) cells, as shown by high recall ratings. However, a significant percentage of early apoptotic cells were misclassified, most frequently as viable (15/25 cases), indicating a systematic bias toward preserved green fluorescence as a marker of cell viability. This suggests that the MLLM over‐relies on dominant color cues and fails to consistently recognize early apoptotic hallmarks such as partial chromatin condensation, subtle membrane blebbing, or mild nucleus‐to‐cytoplasm ratio changes. Late apoptotic cells also exhibited substantial misclassification, being distributed across viable (8/25) and necrotic (7/25) categories, reflecting difficulty in resolving transitional morphologies where apoptotic and necrotic features overlap within the same cell population. Figure 5B represents class‐specific precision, recall, and F1‐scores. The model achieved the highest F1‐score (0.81) for necrotic cells, supported by perfect precision and a recall of 0.68, indicating a comparatively robust ability to identify overt necrotic morphology. In contrast, the viable cells exhibited a high recall (0.84) but substantially lower precision (0.37), resulting in a moderate F1‐score (0.51).

**FIGURE 5:**
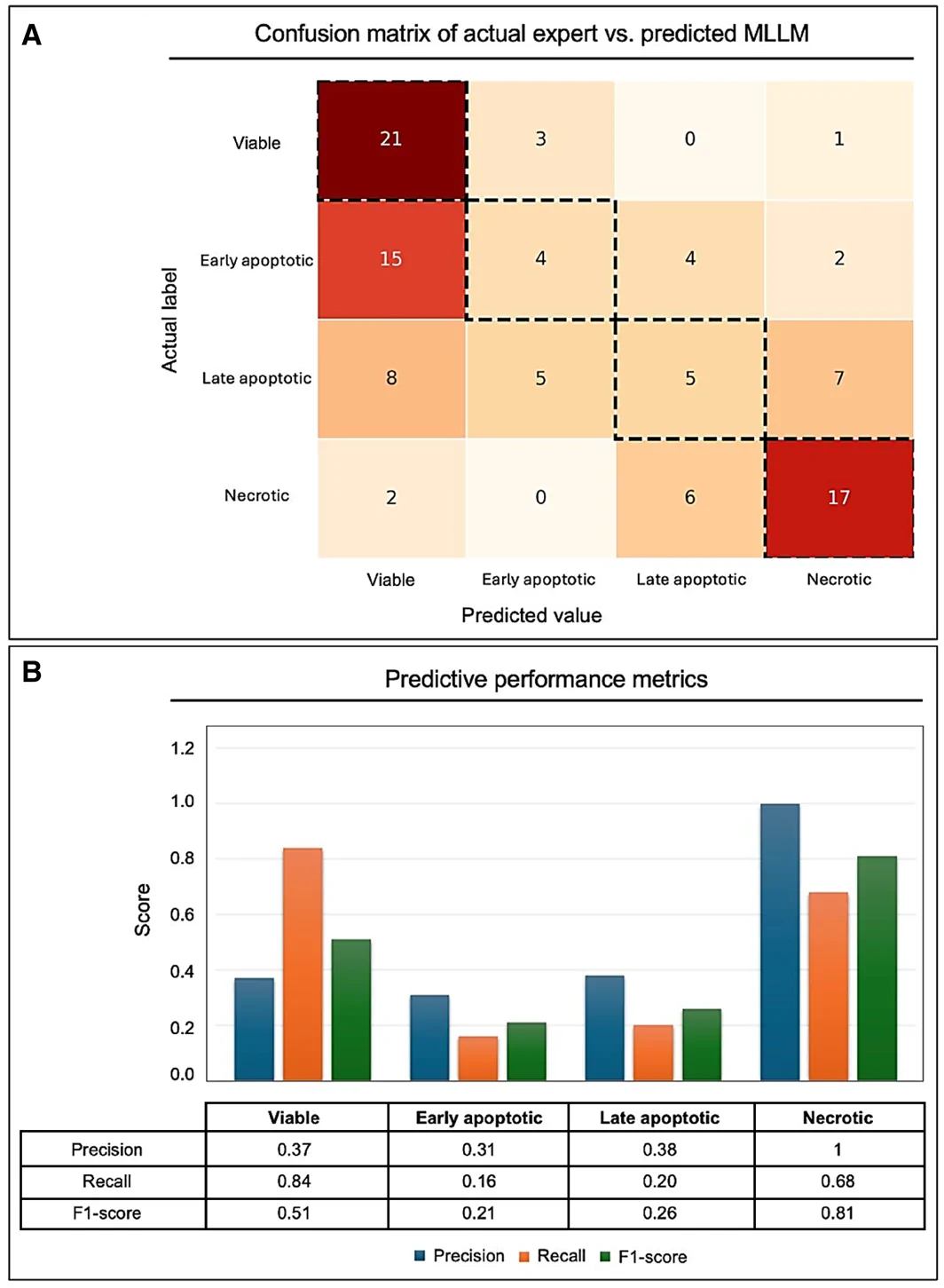
Confusion matrix and predictive performance metrics of MLLM in cell state classification. (A) Confusion matrix comparing MLLM predictions with human expert annotations across four distinct cell states: viable, early apoptotic, late apoptotic, and necrotic. Correctly classified instances are located along the diagonal (highlighted with dashed lines). (B) Bar graph and corresponding table presenting class‐wise precision, recall, and F1‐score values. Although the model shows strong recall for viable and necrotic cells, its performance in identifying early and late apoptotic states remains limited. MLLM, multimodal large language model.

Early apoptotic cells exhibited the lowest recall (0.16) and an F1‐score of only 0.21, while late apoptotic cells demonstrated comparable weakness (recall: 0.20; F1‐score: 0.26). These error patterns indicate that the primary limitation of the MLLM is not the detection of individual visual elements but the inability to integrate multiple concurrent cytopathological cues into a coherent spatial–contextual interpretation. Overall, the results underscore the model’s robust capacity to differentiate between terminal cell states (viable and necrotic), while simultaneously revealing a significant deficiency in accurately recognizing apoptosis.

To further assess the model’s class‐wise discriminative capacity, receiver operating characteristic (ROC) curves and AUC values were computed for each cell state (viable, early apoptotic, late apoptotic, and necrotic), as shown in Figure 6. These metrics provide a threshold‐independent evaluation of classification performance and offer additional insight into the model’s sensitivity–specificity balance.

**FIGURE 6:**
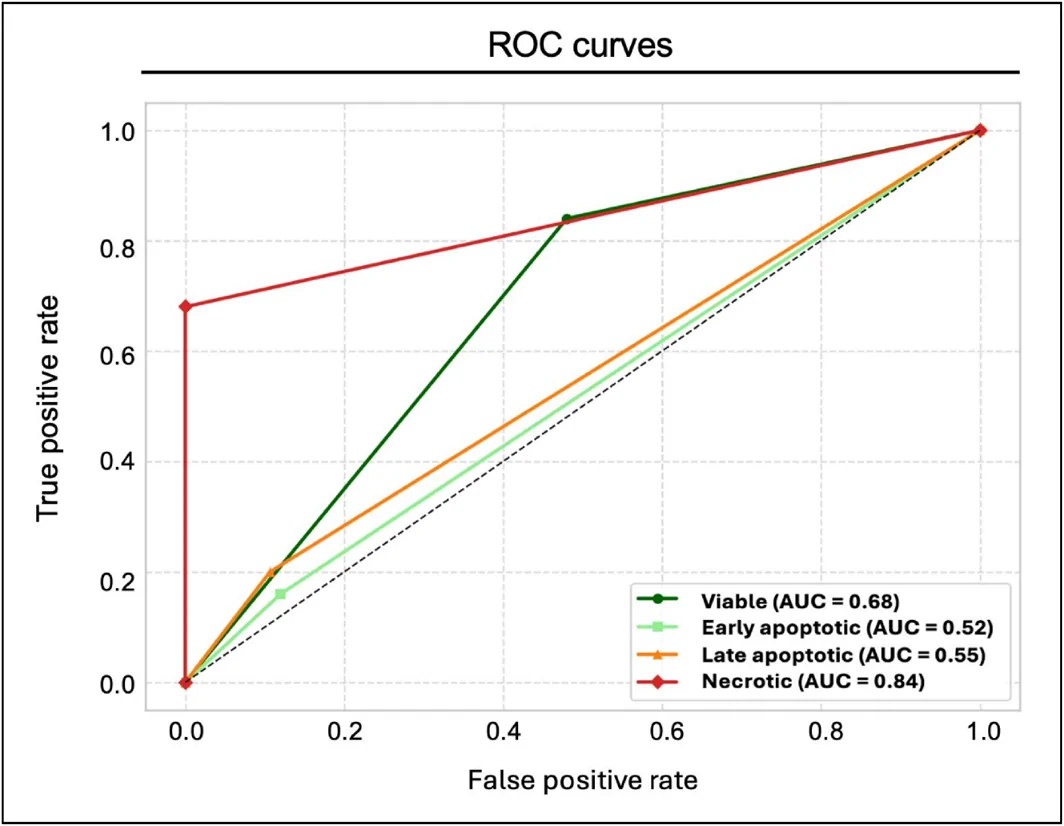
Receiver operating characteristic curves and AUC analysis for cell state classification. The model demonstrates the highest discriminative power for necrotic cells (AUC = 0.84), moderate for viable (AUC = 0.68), and low for apoptotic states (AUC = 0.52 for early apoptotic, AUC = 0.55 for late apoptotic). AUC, area under the curve.

The model achieved its highest AUC for necrotic cells (AUC = 0.84), reinforcing previous findings of reliable necrosis detection. This strong separability suggests that the model effectively identifies overt morphological signatures of necrotic death, such as red‐only nuclear staining and membrane rupture, which are relatively easier to distinguish under AO/PI fluorescence. For viable cells, the AUC was moderate (AUC = 0.68), indicating partial overlap with apoptotic states in the feature space. Although recall was high in earlier metrics, the lower precision and moderate AUC here imply that viable cells are sometimes misclassified when early apoptotic changes are subtle. By contrast, early apoptotic (AUC = 0.52) and late apoptotic (AUC = 0.55) cells demonstrated poor discriminability. These AUC values are only marginally above the random classifier baseline (AUC = 0.50), suggesting that the model struggles to consistently differentiate apoptotic states from others. This aligns with confusion matrix outcomes and F1‐scores, highlighting a persistent challenge in recognizing transient or intermediate morphological cues associated with apoptosis. Taken together, the ROC analysis confirms that although the MLLM can robustly distinguish necrotic cells, its performance in detecting apoptotic transitions remains limited. Enhancing discriminative power in these classes will likely require the integration of higher‐resolution morphological features, temporal context, or multimodal inputs.

## 3 DISCUSSION

The present findings provide a nuanced view of the capabilities and limitations of MLLM‐based cytopathological assessment, particularly when applied to fluorescence‐based evaluation of cell death. It becomes clear that MLLMs are capable of providing high‐throughput assessments with near‐expert accuracy in identifying well‐defined cytopathological features such as TCC and necrosis. For context, manual evaluation of the same 500‐image dataset by three cytopathology‐trained experts—requiring independent review followed by consensus discussion—would be expected to require several tens of human‐hours, highlighting a substantial efficiency gain achieved by the MLLM‐based pipeline. However, their performance declines significantly when it comes to detecting more subtle and transitional apoptotic events. This deficiency becomes particularly evident at intermediate doxorubicin concentrations (e.g., 2.0 μmol/L), where overlapping morphological characteristics of early and late apoptosis make accurate classification more challenging for the model. A comparable shortcoming is also observed in the evaluation of viable cells. Especially at higher drug doses, the model tends to overestimate cell viability. This is likely because of its reliance on the presence of green fluorescence as a marker of viability, without adequately interpreting the subtle nuclear or cytoplasmic changes that occur during the early stages of apoptosis but precede overt cell death. To overcome these limitations, future model development should focus on increasing sensitivity to nuanced apoptotic features such as CC+, NF, and MB. Furthermore, incorporating larger and more diverse training datasets annotated by human experts—particularly those encompassing borderline and transitional morphologies—may significantly improve the model’s reliability and diagnostic value in automated cytopathological workflows. To further interpret these findings at the level of dose‐dependent cytopathological dynamics, it is necessary to examine how apoptotic and necrotic patterns evolve across increasing doxorubicin concentrations.

In line with these observations, examining dose‐dependent apoptotic progression helps explain why model–expert discrepancies emerge most prominently at intermediate and high cytotoxic stress levels. The pronounced peak at 2.0 μmol/L likely reflects a transitional cytotoxic threshold, when a maximal proportion of cells have commenced apoptosis while still exhibiting morphologically detectable early apoptotic characteristics. Elevated doxorubicin concentrations induce rapid advancement of cells into LA or necrosis, leading to structural disintegration and the loss of identifiable early apoptotic morphology as shown using AO/PI fluorescence microscopy. Thus, elevated doses result in diminished early apoptotic indices, not from a decline in apoptotic signaling but because cells have progressed beyond the initial apoptotic phase. This discrepancy at higher concentrations may stem from the model’s limited sensitivity to subtle nuclear changes that characterize early‐stage apoptosis under conditions of widespread cellular damage. Another possible reason could be the visual overlap between advanced apoptotic and necrotic features at this dose, which may have confounded the model’s classification boundaries. Regarding late‐stage apoptotic assessment presented in Figure 2D, human experts successfully identified characteristic features such as NF and MB, with the highest incidence observed at 4.0 μmol/L—where nearly 80% of cells were classified as late apoptotic. In contrast, the MLLM consistently underestimated this category, reporting less than 10% across all doses. This substantial gap suggests that late‐stage apoptosis remains a major challenge for the model, likely due to the irregular morphology of fragmented nuclei and the presence of partially lysed membranes, which can hinder pattern recognition. Another possible reason could be the model’s difficulty in distinguishing late apoptotic cells from necrotic ones, as both may exhibit disrupted nuclear and membrane integrity, leading to overlapping visual cues. This discrepancy reinforces the limitations observed in both early and late apoptotic evaluations, suggesting that the model struggles to integrate distinct apoptotic morphologies into a unified cytopathological interpretation. Another possible reason may involve the model’s limited ability to reconcile transitional features that fall between clearly separable early and late stages, especially when these occur in dense, damaged cellular environments. Unlike apoptotic parameters, the assessment of necrotic cell death revealed a notable consistency between expert evaluations and MLLM outputs as presented in Figure 2F. Both approaches identified a clear dose‐dependent increase in necrotic index (NI %), with only minor variations in quantitative estimates. This close agreement suggests that the model is capable of reliably detecting necrotic morphology, which is typically characterized by intense red nuclear fluorescence and complete loss of membrane integrity. The high visual contrast and reduced morphological ambiguity of necrotic cells may contribute to the model’s comparatively strong performance in this category. Together, these findings indicate that the principal limitation of the MLLM lies not in detecting overt cell death, but in resolving complex and transitional apoptotic morphologies that require integrated spatial and contextual interpretation.

To better explain the mechanistic basis of the observed discrepancies, a morphology‐centered interpretation is required. At a morphological level, these limitations appear to stem not from a failure to detect individual visual elements, but from difficulties in integrating multiple concurrent cytopathological cues into a coherent biological interpretation. In particular, characteristic of transitional apoptosis—such as intermediate chromatin condensation without overt fragmentation, partial membrane permeability accompanied by mixed AO/PI fluorescence, subtle alterations in the nucleus‐to‐cytoplasm ratio, and spatially localized membrane blebbing—were frequently under‐recognized by the MLLM. Although these features are readily interpreted by human experts through contextual and relational reasoning, they require fine‐grained spatial awareness and cross‐feature integration that remain challenging for language‐model‐driven multimodal systems. Importantly, this limitation is not unique to the present model. Similar findings have been observed in other deep learning models developed for label‐free classification of apoptotic and necrotic cells. For instance, the D‐MAINS model achieved > 95% accuracy in detecting apoptotic and necrotic events in HeLa cells without requiring fluorescent markers, while CellDeathPred accurately distinguished between apoptosis and ferroptosis using cell‐stained images [28, 29]. These studies validate the feasibility of morphology‐based AI classification in cell death detection. Consistent with this interpretation, recent cytology‐focused AI studies have demonstrated that high model performance is typically achieved in settings where the target morphology is well‐defined and separable from background structures. For example, a deep learning–based fungal screening model applied to bronchoalveolar lavage cytology achieved a recall of 0.97 by leveraging clearly distinguishable fungal morphologies and explicit background/debris class separation, highlighting the importance of morphological clarity for reliable AI‐assisted cytopathological analysis [30].

In this context, the observed limitations in apoptotic interpretation highlight the critical role of annotation quality and expert‐defined ground truth in shaping AI performance in cytopathological analysis. These observations underscore the importance of expert‐guided annotations in generating reliable ground truth for cytopathological evaluation. A previous report on digital image analysis in immunohistochemistry showed that even the most advanced AI platforms underperform without optimized annotation strategies like region‐specific or hands‐free segmentation [31]. In that study, variability in Ki‐67 scoring was influenced more by the annotation method than by the software platform itself. These insights support the structured approach used in our study, where 15‐parameter cytopathological evaluation checklist were evaluated through a standardized AO/PI‐specific checklist. This methodology ensured consistency across expert and AI assessments, enabling fair and reproducible performance comparisons. Importantly, these findings align with previous reports highlighting the limitations of general‐purpose MLLMs in fine‐grained biomedical image interpretation. Although GPT‐4o has demonstrated promising recall in tasks such as blood morphology classification and fundus image analysis, its precision and domain‐specific sensitivity remain constrained [25, 31]. A recently published editorial report has similarly pointed out ChatGPT’s inconsistent performance in replicating expert‐level segmentation and visually grounded reasoning in medical imaging [32]. Consistent with these observations, a recent benchmark study in cervical cytology reported that, despite promising performance, large language and visual‐language models remain inferior to expert cytopathologists and require substantial methodological refinement before reliable clinical deployment [33]. Taken together, the findings indicate that while the MLLM can accurately reflect human expert judgments in a substantial number of cases, it still demonstrates notable limitations in sensitivity and biological context‐awareness. Improving its ability to detect early‐stage and transitional apoptotic changes, and embedding biologically grounded plausibility mechanisms, remains a critical direction for future development. These considerations provide an important framework for interpreting the present findings and inform future efforts aimed at improving biologically grounded AI‐assisted cytopathological analysis. More broadly, recent cytopathology‐oriented AI studies suggest that such performance asymmetries—where well‐defined morphological targets are detected reliably while transitional cellular states remain challenging, represent a common bottleneck for clinical translation and underscore the need for biologically grounded workflow‐aware model design [34].

In line with these performance trends, the model tends to overpredict viability, particularly in cases where early apoptotic changes are morphologically subtle and partially overlap with viable phenotypes. Consistent with these findings, predictive performance for early and late apoptotic states remained poor, underscoring the model’s limited sensitivity to transitional and intermediate apoptotic features. Future improvements should concentrate on increasing sensitivity to transitional phenotypes, maybe by utilizing enriched datasets and including spatial‐temporal feature recognition modules.

Despite the promising insights provided by the MLLM‐based evaluation, several limitations of the presented study must be acknowledged. For example, a formal quantitative inter‐observer agreement analysis (e.g., Cohen’s kappa) could not be performed, as individual pre‐consensus evaluation scores were not retained, which represents a methodological limitation of this study. In addition, language‐model‐driven architecture lacks spatial perception and domain‐specific priors that are essential for interpreting complex cytomorphological patterns. This constraint likely contributed to the model’s underperformance in detecting transitional apoptotic events, which require not only semantic understanding but also fine‐grained spatial discrimination. Furthermore, the rule‐based conversion of visual patterns into text and back into semi‐quantitative scores may introduce interpretive artifacts that would not arise in end‐to‐end learning frameworks. Looking forward, deep learning–based models—particularly CNNs and transformer‐based vision models—may offer more robust solutions by directly learning hierarchical features from raw image data [35]. These architectures are inherently equipped to capture texture, intensity gradients, and local morphological patterns that define cell viability and death. Eliminating the need for intermediate textual reasoning, deep learning approaches can offer more direct and sensitive analysis of apoptotic morphology, with the added benefit of scalability and high‐throughput capacity [36]. In this context, future improvements may involve structured stepwise prompting strategies in which the model is explicitly guided to identify and describe specific apoptotic hallmarks—such as MB, CC+, or NF—prior to assigning a final cell state, which may help reduce the observed “apoptosis gap” in early and late apoptotic classification. On the other hand, future work should prioritize the integration of large annotated fluorescence datasets with advanced deep learning pipelines to enable context‐aware, biologically grounded, and expert‐comparable cytopathological analysis.

In conclusion, this study demonstrates that while MLLM‐based systems can approximate expert‐level performance in detecting well‐defined terminal cytopathological states, they remain insufficient for resolving biologically complex and transitional apoptotic morphologies. The findings emphasize that reliable AI‐assisted cytopathological interpretation requires not only high‐throughput capability but also biologically grounded contextual reasoning supported by expert‐defined annotation frameworks. Bridging this gap will be essential for the responsible integration of multimodal AI systems into cytopathological research and translational workflows.

## 4 CONCLUSION

This study presents a comparative assessment of expert‐based and AI‐assisted cytopathological evaluation of AO/PI‐stained fluorescence microscopy images, with a focus on distinguishing distinct cell states under doxorubicin‐induced cytotoxic stress. The integration of a MLLM into the analysis pipeline demonstrated encouraging results, with expert‐level concordance observed in over half of the evaluated parameter–dose combinations. The model showed robust performance in identifying viable and necrotic cells, both of which are characterized by well‐defined and morphologically distinct features. However, its consistent underperformance in detecting early and late apoptotic states underscores the limitations of language‐model‐driven architectures in capturing subtle transitional cytological changes. These shortcomings were evident across multiple evaluation layers, including severity scoring, confusion matrices, and ROC analyses. The reliance on indirect textual abstraction appears to restrict the model’s capacity for spatially grounded and biologically nuanced interpretation. Taken together, our findings suggest that while MLLMs hold potential for high‐throughput or pre‐screening applications in fluorescence‐based cell death analysis, more sophisticated approaches are needed to achieve expert‐comparable performance across all morphological states. Deep learning models that operate directly on image data—such as CNNs and transformer‐based vision architectures—offer a promising direction. Their inherent ability to extract hierarchical and context‐rich features without the need for intermediate textual reasoning positions them as suitable candidates for more accurate and sensitive classification of apoptotic and necrotic cell death. Future research should prioritize the development of such models using large well‐annotated fluorescence datasets to advance the automation and reliability of cytopathological image analysis.

## 5 MATERIALS AND METHODS

### 5.1 Materials

MCF‐7 human breast adenocarcinoma cell line was obtained from the American Type Culture Collection (ATCC, HTB‐22^TM^). Dulbecco’s modified eagle medium (DMEM) high glucose, fetal bovine serum (FBS), penicillin–streptomycin solution, phosphate‐buffered saline (PBS), trypsin‐EDTA, and other routine reagents were purchased from Gibco (Thermo Fisher Scientific, USA) unless otherwise specified. Doxorubicin hydrochloride, AO, and PI were obtained from Sigma‐Aldrich (USA). All chemicals used were of analytical grade or cell culture grade.

### 5.2 Cell culture and maintenance

MCF‐7 cells were cultured in DMEM supplemented with 10% FBS and 1% penicillin–streptomycin. Cells were maintained at 37°C in a humidified atmosphere with 5% CO_2_. Subculturing was performed at 70%–80% confluence using trypsin‐EDTA. All experiments were conducted using cells between passage numbers 5 and 8 to ensure cellular consistency.

### 5.3 Anticancer drug exposure

Cells were seeded in 24‐well tissue culture plates at a density of 2 × 10^4^ cells per well and incubated for 24 h to allow for cell attachment. After this period, doxorubicin was added to the wells at final concentrations of 0 μmol/L (control), 0.5 μmol/L, 1.0 μmol/L, 2.0 μmol/L, and 4.0 μmol/L. Each concentration was freshly prepared in culture medium and applied for 24 h under standard incubation conditions.

### 5.4 AO/PI dual staining

After drug exposure, cells were stained with a dual‐staining solution containing 25 μg/mL of AO and 25 μg/mL of PI, each prepared in sterile PBS and filtered through a 0.22 μm syringe filter (Merck Millipore, Germany) prior to use. The staining solution was directly applied to each well for 1 min at room temperature in the dark. After incubation, cells were immediately visualized using a Zeiss Axio Observer A1 fluorescence microscope equipped with standard fluorescein isothiocyanate (FITC) and tetramethylrhodamine isothiocyanate (TRITC) filter sets for detecting green and red fluorescence, respectively.

### 5.5 Image acquisition and dataset structure

Following AO/PI staining, fluorescence microscopy images were acquired under 10× magnification. For each experimental group, five biological replicates were prepared (*n* = 5), and 20 non‐overlapping fluorescence images were captured per well, resulting in 100 images per group and a total of 500 images across all five treatment conditions (control, 0.5 μmol/L, 1.0 μmol/L, 2.0 μmol/L, and 4.0 μmol/L doxorubicin). All images were saved in uncompressed TIFF format to retain high‐resolution fluorescence signal and were systematically labeled according to group and well identifiers. Prior to analysis, the images were anonymized and randomized to minimize observer bias and ensure objective evaluation in both manual and automated analysis phases.

### 5.6 Cytopathological evaluation by researchers

Fluorescence images were independently analyzed by three researchers with training and experience in cytopathology and fluorescence‐based cell imaging. Each image was manually assessed according to a standardized evaluation checklist consisting of four main categories: (1) Total cell counts and density, (2) staining profile and viability, (3) nuclear morphology, and (4) cytoplasmic and membrane morphology. Across these categories, a total of 15‐parameter cytopathological evaluation checklist were systematically evaluated as detailed in Table 1. Manual evaluation was performed using CellProfiler (version 4.2.8, Broad Institute, Cambridge, MA), an open‐source image analysis software platform optimized for high‐throughput fluorescence microscopy. A customized pipeline was designed to facilitate visual verification through segmentation overlays and adjustable display settings, enabling accurate evaluation of nuclear and cytoplasmic features. Region‐specific zooming, threshold adjustment, and classification overlays were used to enhance consistency among evaluators. Each item on the checklist was marked as “observed” or “not observed” per image, and semi‐quantitative estimations were reported as mean ± standard deviation (SD) where applicable (e.g., percentage values). Responses from all three researchers were compiled, and discrepancies were resolved by consensus following joint review of discordant cases. For expert comparison, a total of 500 fluorescence microscopy images (100 per treatment group) were analyzed using the full 15‐parameter cytopathological evaluation checklist. These images were identical to those used in the MLLM evaluation, ensuring consistent comparison. Representative examples from this dataset are displayed in Figure 1. To illustrate the expert consensus process and the inherent complexity of cytopathological interpretation, a representative expert consensus‐based cytopathological assessment corresponding to the same AO/PI‐stained image analyzed by the MLLM is provided in Table S1. Because of the time‐intensive and cognitively demanding nature of detailed multi‐parameter cytopathological annotation, human expert evaluations were intentionally focused on a representative subset of images per treatment group. This approach reflects realistic expert‐annotation constraints in routine cytopathological assessment. In contrast, the MLLM was applied to the full image dataset to evaluate scalability, throughput, and performance under high‐volume analysis conditions.

**TABLE 1:** Checklist of AO/PI image evaluation criteria grouped into five cytopathological categories, as used in expert‐guided analysis.

Category I: Total cell count		
1	Total cell count (TCC)	Approximate number of countable cells per image
Category II: Staining profile and viability		
2	Viable cells index (VI %)	Percentage of viable cells with intact green fluorescence
3	Early apoptotic cell index (EAI %)	Bright green, condensed nuclei, without PI uptake
4	Late apoptotic cell index (LAI %)	Orange to red‐stained fragmented nuclei with partial membrane
5	Total apoptotic index (TAI %)	Total percentage of early and late apoptotic cells
6	Necrotic index (NI %)	Percentage of necrotic cells possessing only red stained nuclei without membrane
7	Acidic vesicular organelles (AVO)	Presence of cytoplasmic acidic vesicular organelles‐stained orange‐green by AO
Category III: Nuclear morphology		
8	Chromatin condensation (CC+)	Dense, non‐fragmented chromatin typical of early apoptosis
9	Nuclear fragmentation (NF)	Fragmented nuclei or apoptotic bodies indicating late apoptosis
10	Nuclear swelling (NS)	Enlarged or diffusely stained nuclei typical of necrosis
Category IV: Cytoplasmic and membrane morphology		
11	Membrane blebbing (MB)	Loss of plasma membrane integrity
12	Blebbing (BB)	Presence of membrane blebs, dynamic and rounded protrusions, cytoskeletal disassembly
13	Cytoplasmic granularity (CG)	Granular cytoplasmic texture indicating organelle or cytoskeletal damage
14	Morphological shift (MS)	Changes in cell shape (e.g., rounding, elongation, shrinkage)
15	Nucleus‐to‐cytoplasm ratio (N/C↓)	Decreased: Enlarged cytoplasm (necrosis)/Increased: Enlarged nucleus (apoptosis)

### 5.7 Cytopathological evaluation via multimodal large language model (LLM)

In parallel with the expert‐based evaluation, all 500 AO/PI‐stained fluorescence microscopy images were assessed utilizing a multimodal LLM deployed via the Poe platform. The selected model, Claude 3.7 Sonnet, supports image input and multimodal reasoning, enabling interpretation of fluorescence‐based cytopathological features. A custom chatbot instance, designated CYTO_ATILIMMED, was developed specifically for this study. The chatbot was configured using a single fixed system‐level instruction prompt that incorporated a standardized 15‐parameter cytopathological evaluation checklist, covering TCC, staining profile and viability, apoptotic and necrotic indices, nuclear morphology, and cytoplasmic and membrane features. The complete instruction prompt, including definitions of all parameters, scoring rules, and the structured output template, is provided in full in Supporting Information to ensure reproducibility and transparency. To minimize stochastic variability while preserving sensitivity to visual patterns, all model evaluations were conducted using a temperature setting of 0.3, promoting deterministic behavior across independent runs. Images were randomized prior to upload to minimize sequence‐related bias. Each image was assessed independently, without access to treatment group information.

The MLLM was instructed to generate structured textual outputs following a predefined schema, comprising (i) qualitative morphological observations and (ii) semi‐quantitative categorical indices. Importantly, the model was not instructed to generate exact numerical percentages. Instead, all quantitative descriptors (e.g., viability, apoptosis, and necrosis) were constrained to predefined ordinal bins: None (0%), Minimal (1%–5%), Moderate (6%–20%), and Extensive (> 20%), consistent with the evaluation framework summarized in Table 1. These values therefore represent direct categorical estimations produced by the model under a fixed rubric, and no secondary quantification or post hoc interpretation of free‐text outputs was performed by the authors.

To assess intra‐model repeatability, a randomly selected subset of 50 images (10 per treatment group) was re‐evaluated after a 72‐h interval using identical prompt and parameter settings. Intra‐model repeatability was defined as consistency in the final four‐class cell state assignment (viable, early apoptotic, late apoptotic, and necrotic), rather than parameter‐wise agreement across the full 15‐parameter cytopathological evaluation checklist. Model outputs were then compiled and compared with expert‐based evaluations described in Section 5.6 to enable assessment of concordance patterns, variability, and systematic deviations between human and MLLM‐based interpretations.

### 5.8 Visual encoding of qualitative assessments for heatmap presentation

To facilitate the visual representation of expert‐assessed cytopathological features across varying doxorubicin concentrations, qualitative descriptors from the evaluation format were systematically converted into numerical values. Specifically, the categories were mapped as follows: “None” (0%) to 0, “Minimal” (1%–5%) to 1, “Moderate” (6%–20%) to 2, and “Extensive” (> 20%) to 3. This quantification enabled the generation of a heatmap using Python’s matplotlib and seaborn libraries, where color gradients correspond to the severity of morphological changes. Both expert‐based and MLLM‐based heatmaps were constructed using a unified color scale (YlOrRd) to ensure direct visual comparability. By standardizing qualitative assessments on a numerical scale, the heatmap provides an intuitive and comparative visualization of dose‐dependent cytopathological alterations.

### 5.9 Quantitative agreement and predictive performance analysis

All quantitative data obtained from both expert evaluations and AI‐assisted image analyses were compiled and analyzed using GraphPad Prism (version 10.4.2) and Python (version 3.11.5). For each treatment group, numerical values—including total cell counts, apoptotic and necrotic indices, and morphological parameters—were expressed as mean ± SD. Group comparisons were performed using one‐way analysis of variance (ANOVA) followed by Tukey’s post hoc test for multiple comparisons. A *p*‐value of less than 0.05 (*p* < 0.05) was considered statistically significant.

To assess the categorical prediction performance of the MLLM, a subset of 100 AO/PI‐stained fluorescence microscopy images was selected. Each image was annotated by expert evaluators based on predominant cytopathological features and assigned one of four class labels: viable, early apoptotic, late apoptotic, or necrotic. The same images were independently evaluated by the MLLM (Claude 3.7 Sonnet, CYTO_ATILIMMED instance), which generated structured predictions aligned with the defined classification scheme. Resulting label pairs (true_label and MLLM_prediction) were compiled into a confusion matrix to assess prediction accuracy. Key performance metrics—including precision, recall, and F1‐score—were calculated for each class using scikit‐learn (version 1.3.2). Furthermore, one‐vs‐rest ROC curves were generated, and AUC values were calculated following label binarization. Cohen’s kappa coefficient was also calculated to quantify categorical agreement between expert annotations and MLLM predictions. All computational analyses were performed in Python using the pandas, scikit‐learn, matplotlib, and seaborn libraries.

## AUTHOR CONTRIBUTIONS

**Atakan Tevlek**: Conceptualization; data curation; formal analysis; funding acquisition; investigation; methodology; project administration; supervision; validation; visualization; writing—original draft; writing—review and editing. **Ozgecan Ocakci**: Data curation; formal analysis; investigation; writing—original draft. **Beyza Emeksiz**: Data curation; investigation; visualization; writing—original draft.

## CONFLICT OF INTEREST STATEMENT

The authors declare no conflicts of interest.

## ETHICS STATEMENT

This study did not involve human participants or animals. All analyses were performed previously generated in vitro fluorescence microscopy images of established cell lines. Therefore, ethical approval and informed consent were not required.

## Supporting information

Supporting Information S1

## Data Availability

The datasets used and analyzed during the current study are available from the corresponding author on reasonable request.
