# Supplementary material for: Artificial intelligence‐enabled cytopathological interpretation of fluorescence microscopy images via a multimodal large language model
Source: Quant Biol. 2026 Apr 30;14(3):e70042. doi: 10.1002/qub2.70042 (PMC13642086; doi:10.1002/qub2.70042)
Supplement: Supplementary file 1 [file QUB2-14-e70042-s001.docx]

**Supplementary File**

**Cytopathological Evaluation Framework and System-Level Instruction Content Used for the CYTO_ATILIMMED Chatbot**

Atakan Tevlek^1,*^, Ozgecan Ocakcı^1^, Beyza Emeksiz^2^

^1^Department of Medical Biology, Faculty of Medicine, Atilim University, 06830, Ankara, Türkiye

^2^Undergraduate Program, Faculty of Medicine, Atilim University, 06830, Ankara, Türkiye

**S1. Overview**

This supplementary file details the cytopathological evaluation framework and the complete system-level instruction content used to configure the custom multimodal large language model (MLLM) chatbot CYTO_ATILIMMED. The framework is based on established acridine orange/propidium iodide (AO/PI) fluorescence microscopy evaluation criteria commonly applied in *in vitro* apoptosis and necrosis studies and was adapted to ensure compatibility with structured AI-based interpretation. The same fixed instruction content was applied consistently to all images analyzed in the study.

**S2. System-Level Instruction Prompt**

You are an expert cytopathology assistant specialized in interpreting **AO/PI dual-stained fluorescence microscopy images** of cultured cells.

Your task is to evaluate each image strictly based on visible cytomorphological features and to report findings using the standardized checklist and scoring framework defined below.

**Constraints:**

- Base all interpretations solely on the visual information present in the image.
- Do not assume treatment group, experimental condition, or time point.
- Avoid speculative biological interpretations beyond AO/PI-based morphology.
- If a parameter cannot be reliably assessed, report it as *Uncertain*.

**AO/PI Morphological Definitions:**

- **Viable cells:** Bright green AO fluorescence, intact nuclear morphology, no PI uptake.
- **Early apoptotic cells:** Condensed or brightly fluorescent green nuclei with preserved membrane integrity.
- **Late apoptotic cells:** Orange to red nuclear staining with fragmentation and partial membrane compromise.
- **Necrotic cells:** Intense red PI-positive nuclei with nuclear swelling and complete membrane breakdown.

**S3. Cytopathological Evaluation Checklist**

Each fluorescence microscopy image was evaluated according to a standardized 15-parameter cytopathological evaluation checklist, which collectively capture cell population characteristics, apoptotic and necrotic progression, and associated nuclear and cytoplasmic morphological features:

1. **Total cell count (TCC)** – Approximate number of countable cells within the field of view.
2. **Viable cells index (VI %)**– Proportion of viable cells exhibiting green fluorescence.
3. **Early apoptotic index (EAI)** – Cells showing condensed or intensely green nuclei with intact membranes.
4. **Late apoptotic index (LAI)** – Cells displaying orange/red nuclear staining with partial membrane compromise.
5. **Total apoptotic index (TAI)** – Combined proportion of early and late apoptotic cells.
6. **Necrotic index (NI)** – Cells exhibiting intense red PI staining and necrotic morphology.
7. **Acidic vesicular organelles (AVO)** – Presence of red AO accumulation in cytoplasmic vesicles.
8. **Condensed chromatin (CC⁺)** – Presence of cells with condensed but unfragmented chromatin.
9. **Nuclear fragmentation (NF)** – Appearance of apoptotic bodies or fragmented nuclei.
10. **Nuclear swelling (NS)** – Enlarged, diffusely stained nuclei indicative of necrotic degeneration.
11. **Membrane breakdown (MB)** – Loss of plasma membrane integrity.
12. **Blebbing (BB)** – Formation of membrane protrusions associated with apoptosis.
13. **Cytoplasmic granularity (CG)** – Alterations or loss of cytoplasmic homogeneity.
14. **Morphological Shift (MS)** – Changes in overall cell shape, including rounding or elongation.
15. **Nucleus-to-cytoplasm ratio (N/C)** – Relative increase (apoptosis) or decrease (necrosis) in nuclear-to-cytoplasmic ratio.

**S4. Scoring Strategy**

For parameters reflecting the proportion of affected cells, the chatbot was not instructed to calculate exact numerical percentages. Instead, evaluations were constrained to predefined semi-quantitative ordinal categories, consistent with common cytopathological practice:

- **None:** 0%
- **Minimal:** 1%–5%
- **Moderate:** 6%–20%
- **Extensive:** > 20%

This categorical approach was intentionally adopted to avoid artificial numerical precision and to reflect the qualitative-to-semi-quantitative nature of fluorescence-based cytopathological assessment.

**S5. Output Structure**

The CYTO_ATILIMMED chatbot generated outputs using a fixed structured format comprising:

- Parameter-wise tabulated evaluations for all 15 criteria
- Semi-quantitative categorical scores
- Brief qualitative observations highlighting key visual cues

All reported values represent **direct model-generated assessments** produced under the fixed instruction framework. No post hoc numerical conversion, recalculation, or manual reinterpretation of the model outputs was performed by the authors.

**S6. Representative example of structured cytopathological output generated by the multimodal LLM (Claude 3.7 Sonnet) under the fixed CYTO_ATILIMMED system prompt**

A representative example of the raw output generated by the CYTO_ATILIMMED chatbot for an AO/PI-stained fluorescence microscopy image is provided in **Supplementary Figure S1**, illustrating the standardized output structure and the direct application of the evaluation framework.

**S7. Expert Consensus-Based Cytopathological Evaluation Framework**

Expert-based cytopathological evaluations were independently performed by three domain experts using the same standardized 15-parameter AO/PI evaluation checklist applied in the AI-based analysis. Each expert initially completed the evaluation in an independent and blind manner.

Following independent scoring, all expert reports were reviewed in a structured panel discussion. Parameters showing disagreement or ambiguity were re-evaluated collectively, and a final consensus decision was reached through discussion of morphological criteria and visual evidence. The resulting consensus-based evaluations were recorded as the definitive expert reference for subsequent comparisons.

To ensure methodological consistency with the multimodal LLM outputs, expert assessments were reported using the same semi-quantitative ordinal categories (None, Low, Moderate, High). Approximate percentage values are provided for illustrative purposes only and reflect expert estimates rather than precise quantitative measurements.

A representative expert consensus-based evaluation corresponding to the same AO/PI-stained image analyzed by the CYTO_ATILIMMED chatbot is provided in Supplementary Table S1, enabling direct qualitative and categorical comparison between expert and AI-based interpretations.

**Supplementary Figure S1.** The model evaluates AO/PI-stained fluorescence microscopy images using a predefined 15-parameter cytopathological checklist. Semi-quantitative indices (e.g., EAI, LAI, NI) are directly inferred by the model based solely on visual morphology, without post-processing or manual quantification.

**Supplementary Table S1.** Expert consensus-based cytopathological evaluation of a representative AO/PI-stained fluorescence microscopy image (Image code: 1C75). Evaluations were independently performed by three experts using the same 15-parameter checklist applied in the AI-based analysis, followed by panel-based consensus. Percentage values are approximate and provided for illustrative purposes only; comparisons were conducted using semi-quantitative ordinal categories.

**Expert consensus-based cytopathological evaluation summary**

**Image code: 1C75**

| **No** | **Abbreviation** | **Definition** | **Explanation** |
| --- | --- | --- | --- |
| 1 | TC | Total cell count | 47 |
| 2 | AO+ | Acridine orange positive | %46,80 (High) |
| 3 | EAI | Early apoptotic index | %17,02 (Moderate) |
| 4 | LAI | Late apoptotic index | %27,65 (High) |
| 5 | TAI | Total apoptotic index | %44,68 (High) |
| 6 | NI | Necrotic index | %8,51 (Moderate) |
| 7 | AVO | Acidic vesicular organelles | %2,12 (Low) |
| 8 | CC+ | Condensed chromatin positive | %17,02 (Moderate) |
| 9 | NF | Nuclear fragmentation | %12,76 (Moderate) |
| 10 | NS | Nuclear swelling | %8,51 (Moderate) |
| 11 | MB | Membrane breakdown | %33,33 (High) |
| 12 | BB | Blebbing | %10,63 (Moderate) |
| 13 | CG | Cytoplasmic granularity | %17,02 (Moderate) |
| 14 | MS | Morphological shift | %27,65 (High) |
| 15 | S + C/N | Swelling + nucleus-to-cytoplasm ratio ↑ or ↓ | Most cells show a relatively preserved N/C ratio with well-defined nuclei |

Semi-quantitative categorization used for expert interpretation:

•	None = 0% •	Low = 1%–5% •	Moderate = 6%–20% •	High = >20%

Expert evaluations were recorded using the same semi-quantitative ordinal categories (None, Low, Moderate, High) applied in the AI-based analysis, to ensure methodological consistency and comparability. Reported percentage values reflect approximate expert-based counts and are provided for illustrative purposes only; all comparative analyses were performed using the ordinal categories.
